# Taxonomic signal in the wing cells of *Lutzia mosquitoes* (Diptera: Culicidae) in Thailand: An outline-based geometric morphometric approach

**DOI:** 10.5455/javar.2025.l916

**Published:** 2025-06-02

**Authors:** Tanawat Chaiphongpachara, Tanasak Changbunjong, Sedthapong Laojun

**Affiliations:** 1 Department of Public Health and Health Promotion, College of Allied Health Sciences, Suan Sunandha Rajabhat University, Bangkok, Thailand; 2 Department of Pre-Clinic and Applied Animal Science, Faculty of Veterinary Science, Mahidol University, Salaya, Thailand; 3 The Monitoring and Surveillance Center for Zoonotic Diseases in Wildlife and Exotic Animals (MoZWE), Faculty of Veterinary Science, Mahidol University, Salaya, Thailand

**Keywords:** Taxonomy, geometric morphometrics, wing shape variation, species identification, outline-based geometric morphometrics

## Abstract

Recent studies have identified wing cells, a substructure of the wing, as carrying taxonomic signals across various mosquito genera. However, the presence of a taxonomic signal in the wing cells of the genus *Lutzia* (Diptera: Culicidae) has not yet been confirmed.

**Objective:**

This study aimed to evaluate the efficacy of the outline-based geometric morphometric (GM) approach in distinguishing *Lutzia* species found in Thailand, namely *Lutzia chiangmaiensis*, *Lutzia fuscana*, *Lutzia halifaxii,* and *Lutzia vorax*.

**Materials and Methods:**

The outline-based geometric morphometrics was employed to analyze four wing elements: the wing contour, the second submarginal cell, the first posterior cell, and the third posterior cell.

**Results:**

In the size analysis, *Lt*. *vorax* consistently exhibited significantly larger wing elements compared to the other species (*p* < 0.05). The factor maps based on discriminant analysis for the wing elements among the species indicated that most groups overlapped in morphospace. However, for the third posterior cell, the *Lt*. *vorax* group presented a more distinct shape. While shape analysis detected significant differences between almost all species pairs (*p* < 0.05), there was an exception between *Lt*. *halifaxii* and *Lt*. *chiangmaiensis* in the first posterior cell (*p* > 0.05). Additionally, shape analysis further indicated that the third posterior cell achieved the highest percentage of correct classifications, with an adjusted total assignment accuracy of 71%.

**Conclusion:**

This finding reveals a significant taxonomic signal in the third posterior cell, suggesting that the outline-based GM approach can effectively complement the landmark-based GM approach in distinguishing *Lutzia* species.

## Introduction

*Lutzia* mosquitoes are large mosquitoes in the family Culicidae and the subfamily Culicinae. Globally, the genus comprises nine species divided into three subgenera: *Insulalutzia* (containing one species), *Lutzia* (two species), and *Metalutzia* (six species) [1,2]. A distinctive characteristic of adult *Lutzia* mosquitoes is the presence of four or more lower mesepimeral setae, setting them apart from other members of the tribe Culicini [1]. The larvae of *Lutzia* are typically found in various groundwater environments and may also inhabit tree holes [1,3]. These larvae are also frequently found in man-made containers and appear to either tolerate or prefer organically rich water [3]. Interestingly, all species within the genus are predaceous during the larval stage, primarily consuming other mosquito larvae and various insects [4]. Adult females generally feed on the blood of mammals and birds, including domestic animals and occasionally on humans [1,3]. *Lutzia* mosquitoes are not known to transmit human pathogens; they play a crucial role in controlling the populations of other mosquito species and may potentially contribute to the biological control of vectors that transmit pathogens. Previous studies have demonstrated the value of *Lutzia* mosquitoes as predators, particularly in reducing the populations of mosquito vectors, such as *Aedes aegypti* (L.), a major vector of dengue [3,5].

In Thailand, four *Lutzia* species, all belonging to the subgenus *Metalutzia*, have been reported: *Lutzia chiangmaiensis* Somboon and Harbach, [6]; *Lutzia fuscana* (Wiedemann, 1820); *Lutzia halifaxii* (Theobald, 1903); and *Lutzia vorax* Edwards, 1921 [3,6]. *Lutzia fuscana, Lt. halifaxii*, and *Lt. vorax* are widespread throughout the country, whereas *Lt. chiangmaiensis* is found in the northern, eastern, and western regions [3,6,7]. Despite their prevalence across Thailand, knowledge about the biology and ecology of these mosquitoes remains limited. This knowledge gap partly arises because accurate identification relies heavily on morphological features, a process often prone to errors, particularly when specimens are damaged [8,9].

In biological classification, taxonomic rank indicates the position of organisms within a hierarchical structure that reflects their evolutionary relationships. Traditional insect taxonomic identification generally involves analyzing and measuring morphological features, supplemented by using taxonomic keys to assign organisms to categories such as family, genus, or species [9]. Although this traditional method is widely used and relatively inexpensive, it becomes challenging when specimen quality is poor and also requires highly skilled taxonomists, who are limited in number in Thailand [9]. Currently, molecular techniques have gained popularity for their high accuracy in identifying species that are difficult to distinguish by morphological methods, including mosquitoes [10,11]. A recent report indicates that molecular methods, such as DNA barcoding, are effective in identifying *Lutzia* mosquitoes [7]. However, these molecular techniques come with limitations, primarily the high costs associated with investigating large numbers of field-collected mosquitoes. Therefore, it is crucial to explore alternative, cost-effective techniques, especially for those with limited budgets.

Geometric morphometrics (GM) is a technique that uses mathematical representations of biological forms based on geometric definitions of their size and shape [12,13]. This technique has been applied to mosquitoes in various research aspects, including intraspecific and interspecific variation, parasite detection, sexual dimorphism, and morphological plasticity [14]. Additionally, GM has been effectively applied to identify various insect vectors, including black flies [15], horse flies [16], *Stomoxys* flies [17], and mosquitoes [18–20]. Insect wings are typically selected for GM analysis because they provide a clear, two-dimensional plane enriched with species-specific characteristics [14]. Notably, this technique has significant advantages in that it is inexpensive, requires only basic scientific equipment, and allows for rapid analysis [21,22]. The landmark-based GM approach is favored for its straightforward process of defining true anatomical landmarks that cover the entire wing‘s morphology. True anatomical landmarks are considered homologous, meaning they occupy equivalent positions across different organisms [23]. In this context, homology refers to the positional equivalence of specific biological structures, which may be precise down to individual points at the relevant scale. The degree of landmark homology is determined by the accuracy and consistency with which landmarks can be identified across organisms [23].

A recent study by Laojun et al. [7] demonstrated that landmark-based GM effectively distinguishes the four *Lutzia* species in Thailand. However, the landmark-based GM approach has a limitation in that it requires the overall wings to be intact and undamaged. In contrast, assessing wing substructures, such as wing cells, using the outline-based GM approach may overcome this limitation when wing samples are incomplete.

Studies using the outline-based GM approach have explored taxonomic signals in wing cells in several mosquito genera, including *Aedes, Anopheles, Armigeres*, *Culex*, and *Mansonia* [24,25]. The outline-based GM method employs pseudo-landmarks to characterize contours or boundary outlines of the target element. These pseudo-landmarks differ from true landmarks in that they are not expected to be comparable individually; rather, their value lies in the structure they collectively describe [25]. However, using the outline-based GM approach to find the presence of a taxonomic signal in the wing cells of *Lutzia* mosquitoes has not been investigated.

To investigate the taxonomic signal in the wing cells of *Lutzia* mosquito species, this study aimed to evaluate the efficacy of the outline-based GM approach in distinguishing *Lutzia* species found in Thailand, namely *Lt*. *chiangmaiensis, Lt*. *fuscana, Lt*. *halifaxii,* and *Lt*. *vorax.* Our analysis focused on four wing elements: the wing contour, the second submarginal cell, the first posterior cell, and the third posterior cell. These wing elements were selected based on previous studies that effectively identified taxonomic signals within these specific structures across various mosquito species [23,25]. The findings from this study provide valuable insights that aid in identifying these mosquitoes based on their wing cell geometry.

## Materials and Methods

### Ethical approval

This study was conducted in accordance with animal care and use guidelines. The research protocol was reviewed and approved by the Institutional Animal Care and Use Committee (IACUC) of Suan Sunandha Rajabhat University, Thailand, with the ethics approval number IACUC 64–007/2021.

### Mosquito collection and morphological identification

Adult *Lutzia* mosquitoes are difficult to capture with light traps [3]; therefore, larvae were collected using the standard dipping method with a mosquito scoop from breeding sites in several regions of Thailand (Table 1) between June 2021 and April 2022. The breeding grounds for these mosquitoes typically include containers with stagnant water in residential or garden areas and small puddles adjacent to rice fields [3].

**Table 1.** Lutzia specimens used in the study and details of their collection sites. Elevation data were taken from the website mapcoordinates.net at https://www.mapcoordinates.net/.

Collection sites (province, district, sub-district)	GIS coordinates	Regions in Thailand	Elevation (m)	Number of specimens per *Lutzia* species			
				*Lt. chiangmaiensis*	*Lt. fuscana*	*Lt. halifaxii*	*Lt. vorax*
Kanchanaburi, Sai Yok, Bongti	14°06'02.5"N, 99°00'01.1"E	Western	233	10	0	0	3
Ratchaburi, Suan Phueng, Thanao Si	13°22'34.0"N, 99°16'26.0"E	Western	225	5	9	0	10
Ranong, Mueang Ranong, Koh Phayam	9°50'28.5"N, 98°27'15.6"E	Southern	34	0	0	10	0
Ubon Ratchathani, Buntharik, Huai Kha	14°34'38.6"N, 105°21'41.2"E	Northeastern	182	5	0	0	3
Chachoengsao, Tha Takiap, Tha Takiap	13°27'14.0"N, 101°46'25.5"E	Eastern	84	5	29	0	4
Trat, Koh Chang, Koh Chang	12°08'27.9"N, 102°16'35.1"E	Eastern	54	0	3	0	2
Trat, Koh Kood, Koh Chang	11°38'16.3"N, 102°33'23.0"E	Eastern	38	5	0	25	0
Total				30	41	35	22

*Lutzia* larvae from each breeding site were placed into white plastic trays (˜20 larvae per tray) and transported to the laboratory of the College of Allied Health Sciences at Suan Sunandha Rajabhat University, Samut Songkhram Campus, Thailand. Upon arrival at the laboratory, the larvae were reared in the same trays under conditions maintained at 25°C–28°C, with a 12 h light-dark cycle and 50%–60% relative humidity. *Aedes* mosquito larvae from the field served as food for the *Lutzia* larvae. Upon pupation, the pupae were transferred to small plastic cups and placed in mosquito cages with dimensions 30 × 30 × 30 cm. Adult mosquitoes, approximately 3-5 days old, were euthanized by freezing at –20°C. Subsequently, female adult *Lutzia* mosquitoes were morphologically identified under a Nikon SMZ 800 N stereomicroscope (Nikon Corp., Tokyo, Japan) using the illustrated taxonomic keys to the *Lutzia* mosquitoes in Thailand [3].

### Wing preparation and outline digitization

**Figure 1.**
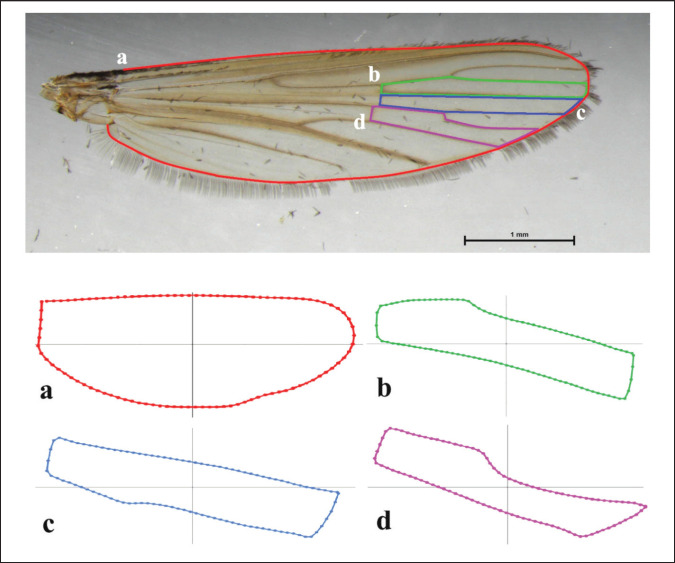
Wing elements of *Lutzia* species used in this study: (a) wing contour; (b) second submarginal cell; (c) first posterior cell; and (d) third posterior cell.

After morphological identification, *Lutzia* specimens with an intact, undamaged right wing were chosen for GM analysis. Each selected specimen was positioned under a stereoscope to excise the right wing from the thorax, which was then mounted on a slide using Hoyer‘s mounting medium. The prepared wing slides were photographed using a Nikon DS-Fi3 digital camera (Nikon Corp., Tokyo, Japan) attached to a Nikon SMZ 800 N stereomicroscope. Each image of the right wing included a scale bar (1 mm) and was assigned an identification number to minimize errors. Four wing elements (Fig. 1)—the wing contour, the second submarginal cell, the first posterior cell, and the third posterior cell—were digitized for GM analysis. These wing elements were selected based on prior studies that identified taxonomic signals across different mosquito species [24,25]. The same set of wing images was consistently used to analyze all four elements.

### Repeatability test

To estimate the digitizing precision of each wing element, the measurement error was evaluated using the repeatability index, as described by Arnqvist and Martensson [26]. This index assesses the shape variance between two measurements. The evaluation was performed using two sets of images from the same sample, which included 20 specimens, randomly selected with five from each *Lutzia* species. These images were digitized twice by the same user, and the resulting repeatabilities were calculated.

### Allometry

The relationship between size and shape, referred to as allometry, was assessed using the linear determination coefficient (*r²*) after regressing the principal components (PCs) of the normalized elliptic Fourier (NEF) coefficients, which represent shape data, against the semimajor axis of the initial ellipse, representing size data, for each wing element. The study of allometry was conducted to determine how variations in wing size might influence wing shape among species and to consider the implications of these variations for evolutionary processes. This exploration is critical, as developmental phases in organisms are generally marked by significant growth, and evolutionary diversification frequently manifests as size differentiation among related taxa [27].

### Size analysis

Half the major axis of the first ellipse, which includes data on the perimeter and the square root of the contour area [28], was used to estimate the size for analyzing wing elements among the four *Lutzia* species. Quantile box plots were generated to illustrate the variation in the global size of these elements across the species. Statistical comparisons of the size among the species within each wing element were performed by one-way analysis of variance (one-way ANOVA). The statistical significance of the one-way ANOVA was estimated by a non-parametric procedure (1,000 replicates). The significance threshold for all statistical analyses in this study was *p* < 0.05.

Additionally, a cross-validated classification, also known as the jackknife procedure, was employed for maximum likelihood classification based on global size [29]. Cross-validation is a predictive assessment technique that evaluates the classification accuracy by considering both expected correct classifications and observed errors in the entire sample set. In this approach, each mosquito was sequentially removed from the total sample, and the removed individual was then classified into the group to which the size was most similar. Finally, the total accuracy score was adjusted for prior probabilities to calculate the adjusted total assignment accuracy. This adjustment accounts for the number of correct assignments that occurred by chance, thereby enhancing the measure of classification effectiveness [30].

### Shape analysis

Elliptic Fourier analysis was employed to define shape variables. This method decomposes contours into sine and cosine curves at successive frequencies, called harmonics, each characterized by four coefficients [31]. NEF coefficients, invariant to size, rotation, and orientation, were calculated based on parameters from the first harmonic ellipse. The fourth coefficient, representing the width-to-length ratio, was retained for further analysis [31]. To reduce the large number of resulting variables, NEF coefficients were subjected to PC analysis (PCA). The PCs obtained served as the final shape variables, which were subsequently used in discriminant analysis (DA) to evaluate group classification among the four Lutzia species. The group classifications were visualized through factor maps based on the first (DF1) and second (DF2) discriminant factors. The number of PCs used for DA was automatically limited to those required to capture 99% of the shape variation (here, 18 PCs). Additionally, Mahalanobis distances between species pairs, derived from DA, were calculated to quantify their dissimilarities.

The statistical significance of these distances was evaluated using a non-parametric permutation-based test with 1,000 replicates and a Bonferroni correction. Similar to the size analysis, a cross-validated reclassification test based on Mahalanobis distances was performed to assess the accuracy of classification. Moreover, Mahalanobis distances between average group shapes were employed to construct the Unweighted Pair Group Method with Arithmetic Mean (UPGMA) trees [28]. These trees depict the shape similarities among the species within each wing element.

### Software

All GM analyses in this study were performed using the online tool XY Online Morphometrics (XYOM) version 3 [28]. This application is freely accessible at https://xyom.io/ and was accessed on December 20, 2024.

## Results

A total of 128 *Lutzia* mosquitoes representing four species were used for GM analyses: 30 *Lt*. *chiangmaiensis*, 41 *Lt*. *fuscana*, 35 *Lt*. *halifaxii*, and 22 *Lt*. *vorax*.

### Repeatability

The measurement error in digitizing wing elements was low, ranging from 4% to 8% for different shapes: 8% for the wing contour, 4% for the second submarginal cell, 4% for the first posterior cell, and 6% for the third posterior cell. These results indicate that the digitizing process in our study was highly accurate, with measurement errors remaining low across all examined wing elements.

### Allometric effect

**Figure 2.**
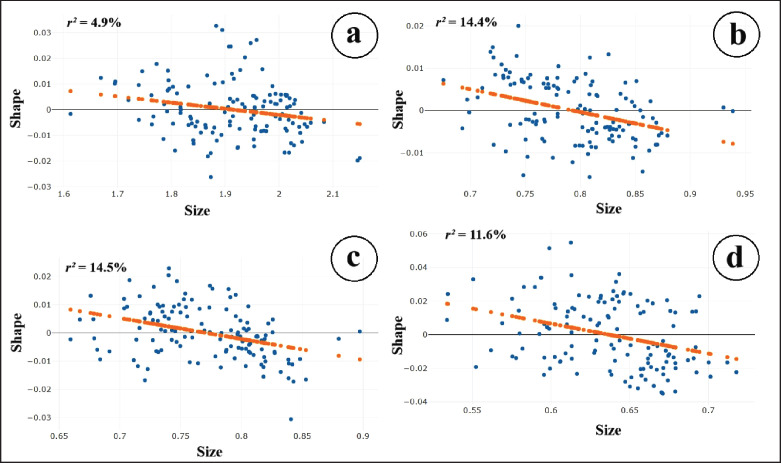
Wing allometric results, showing the relationships between the shape and size of four *Lutzia* species examined in this study for each wing element: (a) wing contour; (b) second submarginal cell; (c) first posterior cell; and (d) third posterior cell. Evaluations from the linear regression for predictions are indicated by orange dots, while blue dots represent individual samples.

The allometric relationships between the shape and size of the four wing elements of the *Lutzia* mosquitoes are depicted in Figure 2. All wing elements demonstrated a negative correlation, a relationship in which one variable increases as the other decreases, and vice versa. This indicates that wing size decreases as shape variation increases, or conversely, wing size increases as shape variation decreases. The coefficients were as follows: *r²* = 4.9% for the wing contour; *r²* = 14.4% for the second submarginal cell; *r²* = 14.5% for the first posterior cell; and *r²* = 11.6% for the third posterior cell. Although size–shape relationships were observed across all wing elements, the allometric effects were not removed from the study because allometric variation is considered integral to evolutionary divergence among taxa.

### Size

**Figure 3.**
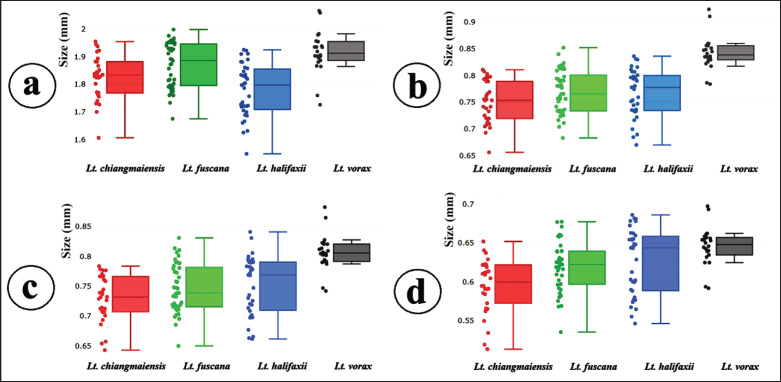
Boxplots illustrating size variation among four *Lutzia* species based on four wing elements: (a) wing contour; (b) second submarginal cell; (c) first posterior cell; and (d) third posterior cell. The horizontal line within each box represents the median, separating the lower 25th percentile from the upper 75th percentile.

In the size analysis, Figure 3 displays the variation in the size of wing elements among the four *Lutzia* species. *Lutzia vorax* consistently exhibited significantly larger wing elements compared to the other species (*p* < 0.05). Size differences among the remaining species varied across different wing elements (Table 2). Additionally, the cross-validated classification based on size among the species revealed very low adjusted total assignment accuracy for the wing elements: 19% for the wing contour; 12% for the second submarginal cell; 13% for the first posterior cell; and 12% for the third posterior cell (Table 3).

**Table 2.** Average wing element size and statistical differences among the four *Lutzia* species.

*Lutzia* species	*n*	Mean size (mm)	Min–Max	Variance	SD
Wing contour					
*Lt. chiangmaiensis*	30	1.825 ^a,b^	1.606–1.955	0.006	0.079
*Lt. fuscana*	41	1.868 ^a,c^	1.675–1.999	0.007	0.082
*Lt. halifaxii*	35	1.778 ^b^	1.548–1.925	0.009	0.096
*Lt. vorax*	22	1. 916 ^c^	1.726–2.067	0.006	0.077
Second submarginal cell					
*Lt. chiangmaiensis*	30	0.750 ^a^	0.656–0.811	0.002	0.040
*Lt. fuscana*	41	0.769 ^a^	0.683–0.852	0.002	0.041
*Lt. halifaxii*	35	0.767 ^a^	0.670–0.836	0.002	0.044
*Lt. vorax*	22	0.842 ^b^	0.784–0.924	0.001	0.032
First posterior cell					
*Lt. chiangmaiensis*	30	0.729 ^a^	0.643–0.784	0.001	0.038
*Lt. fuscana*	41	0.746 ^a^	0.650–0.831	0.002	0.041
*Lt. halifaxii*	35	0.751 ^a^	0.662–0.841	0.002	0.049
*Lt. vorax*	22	0.807 ^b^	0.742–0.882	0.001	0.031
Third posterior cell					
*Lt. chiangmaiensis*	30	0.595 ^a^	0.513–0.652	0.001	0.035
*Lt. fuscana*	41	0.620 ^b^	0.535–0.677	0.001	0.032
*Lt. halifaxii*	35	0.626 ^b,d^	0.547–0.686	0.002	0.042
*Lt. vorax*	22	0.646 ^d^	0.592–0.697	0.001	0.025

Different lowercase letters within a column among *Lutzia* species for each wing element denote statistically significant differences (*p* < 0.05). *n* = sample size; Min = minimum; Max = maximum; SD = standard deviation.

**Table 3.** Percentage of correct identification based on the crossvalidated classification based on the size of the four wing elements in four *Lutzia* species.

*Lutzia* species	No. of correctly assigned samples/No. of total observed samples	Correct assignment scores (%)
Wing contour		
*Lt. chiangmaiensis*	10/30	33.33
*Lt. fuscana*	6/41	14.63
*Lt. halifaxii*	19/35	54.29
*Lt. vorax*	16/22	72.73
Total correct assignment score	51/128	39.84
Adjusted total assignment accuracy		19
Second submarginal cell		
*Lt. chiangmaiensis*	17/30	56.67
*Lt. fuscana*	4/41	9.76
*Lt. halifaxii*	4/35	11.43
*Lt. vorax*	20/22	90.91
Total correct assignment score	45/128	35.16
Adjusted total		12
First posterior cell		
*Lt. chiangmaiensis*	14/30	46.67
*Lt. fuscana*	5/41	12.20
*Lt. halifaxii*	8/35	22.86
*Lt. vorax*	19/22	86.36
Total correct assignment score	46/128	35.94
Adjusted total assignment accuracy		13
Third posterior cell		
*Lt. chiangmaiensis*	16/30	53.33
*Lt. fuscana*	9/41	21.95
*Lt. halifaxii*	6/35	17.14
*Lt. vorax*	14/22	63.64
Total correct assignment score	45/128	35.16
Adjusted total assignment accuracy		12

### Shape

**Figure 4.**
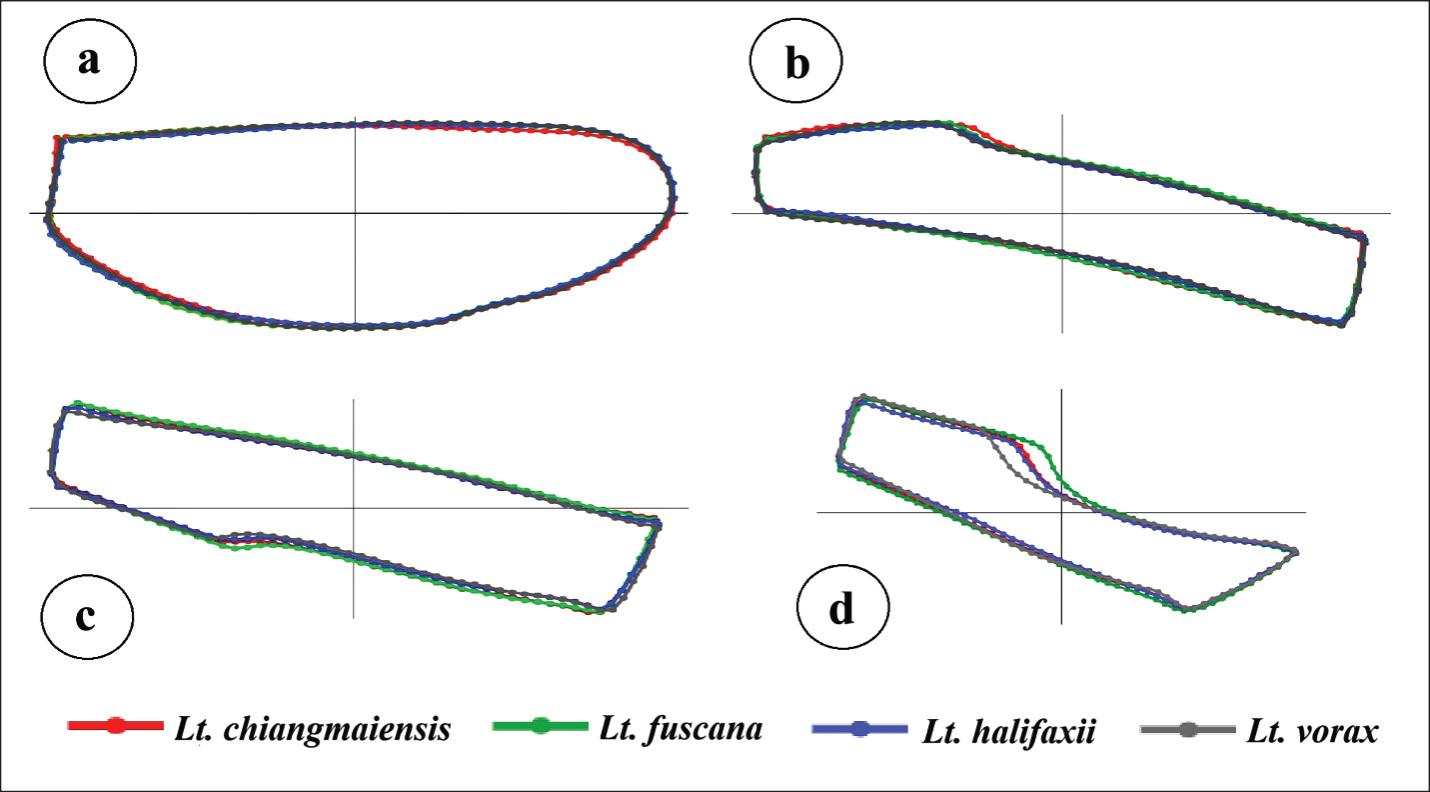
Superpositions of mean shapes illustrating shape differences in wing elements among four *Lutzia* species: (a) wing contour; (b) second submarginal cell; (c) first posterior cell; and (d) third posterior cell.

**Figure 5.**
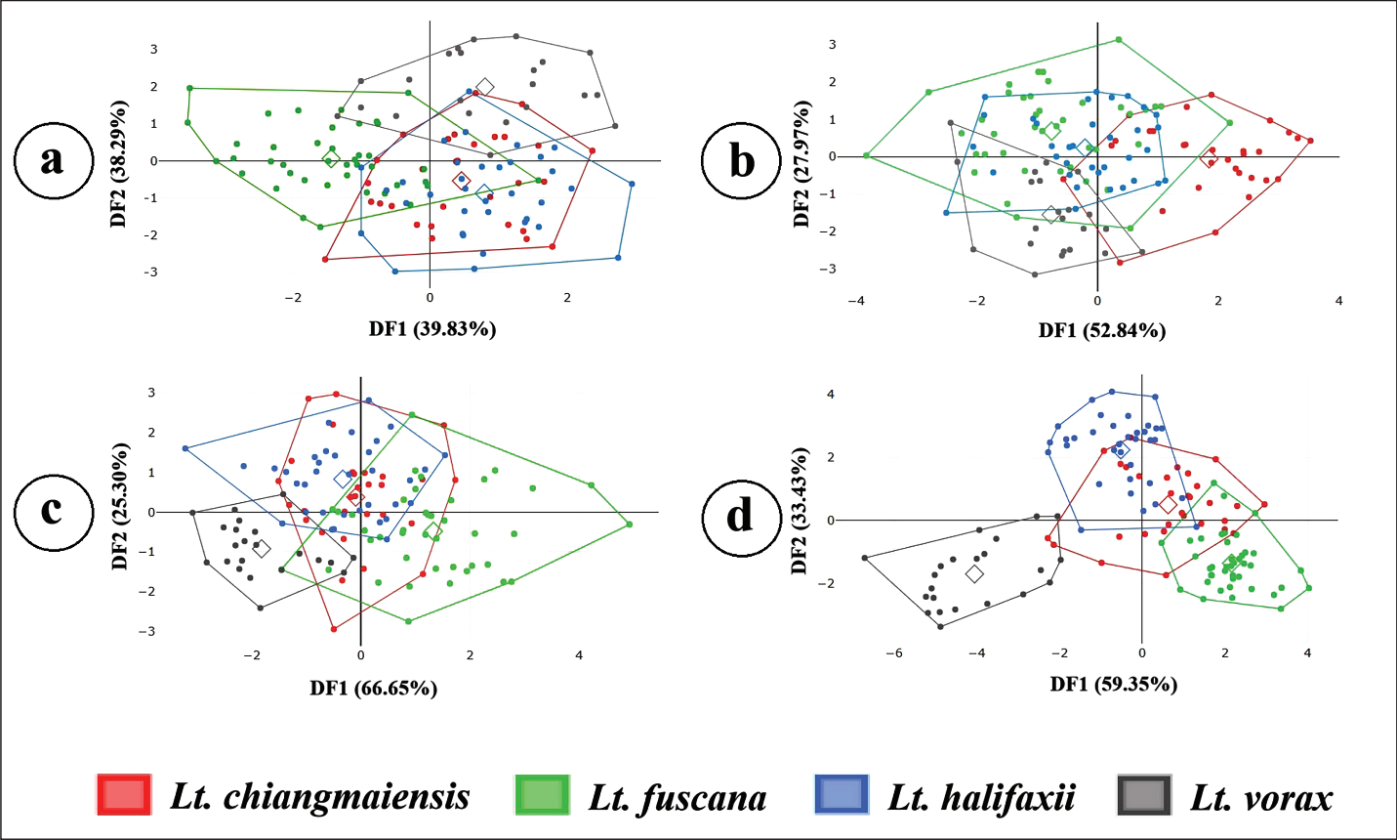
Factor maps illustrating group classification among four *Lutzia* species based on wing shape using the first (DF1) and second (DF2) discriminant factors for four wing elements: (a) wing contour, (b) second submarginal cell, (c) first posterior cell, and (d) third posterior cell.

The superposition of mean shapes across different wing elements revealed no notable differences among species for most elements (Fig. 4). However, the third posterior cell exhibited distinct shape variations, particularly in the upper curvature (Fig. 4d). Discriminant analyses identified three discriminant factors, with the first two discriminant factors accounting for 78.12% of the total variation in wing contour (DF1 = 39.83%, DF2 = 38.29%), 80.81% in the second submarginal cell (DF1 = 52.84%, DF2 = 27.97%), 91.95% in the first posterior cell (DF1 = 66.65%, DF2 = 25.30%), and 92.78% in the third posterior cell (DF1 = 59.35%, DF2 = 33.43%). The factor maps based on DF1 and DF2 for the wing elements among the species indicated that most groups overlapped in morphospace (Fig. 5). However, for the third posterior cell, the *Lt*. *vorax* group presented a more distinct shape (Fig. 5d). Significant differences in Mahalanobis distances among *Lutzia* species across each wing element are presented in Table 4. Almost all species pairs showed significant differences (*p* < 0.05), except for the pair of *Lt*. *halifaxii* and *Lt*. *chiangmaiensis* in the first posterior cell (*p* > 0.05).

**Table 4.** Pairwise Mahalanobis distances and significant differences among four *Lutzia* species based on the shape of four wing elements.

*Lutzia* species	*Lt. chiangmaiensis*	*Lt. fuscana*	*Lt. halifaxii*	*Lt. vorax*
Wing contour				
*Lt. chiangmaiensis*	0			
*Lt. fuscana*	2.413^*^	0		
*Lt. halifaxii*	2.106^*^	2.516^*^	0	
*Lt. vorax*	2.882^*^	2.959^*^	2.949^*^	0
Second submarginal cell				
*Lt. chiangmaiensis*	0			
*Lt. fuscana*	2.728^*^	0		
*Lt. halifaxii*	2.422^*^	1.72^*^	0	
*Lt. vorax*	3.012^*^	2.266^*^	2.231^*^	0
First posterior cell				
*Lt. chiangmaiensis*	0			
*Lt. fuscana*	1.823^*^	0		
*Lt. halifaxii*	1.142	2.133^*^	0	
*Lt. vorax*	2.278^*^	3.178^*^	2.318^*^	0
Third posterior cell				
*Lt. chiangmaiensis*	0			
*Lt. fuscana*	2.968^*^	0		
*Lt. halifaxii*	2.802^*^	4.492^*^	0	
*Lt. vorax*	5.340^*^	6.236^*^	5.325^*^	0

Asterisk indicates a significantly different (*p* < 0.05) species pair.

**Figure 6.**
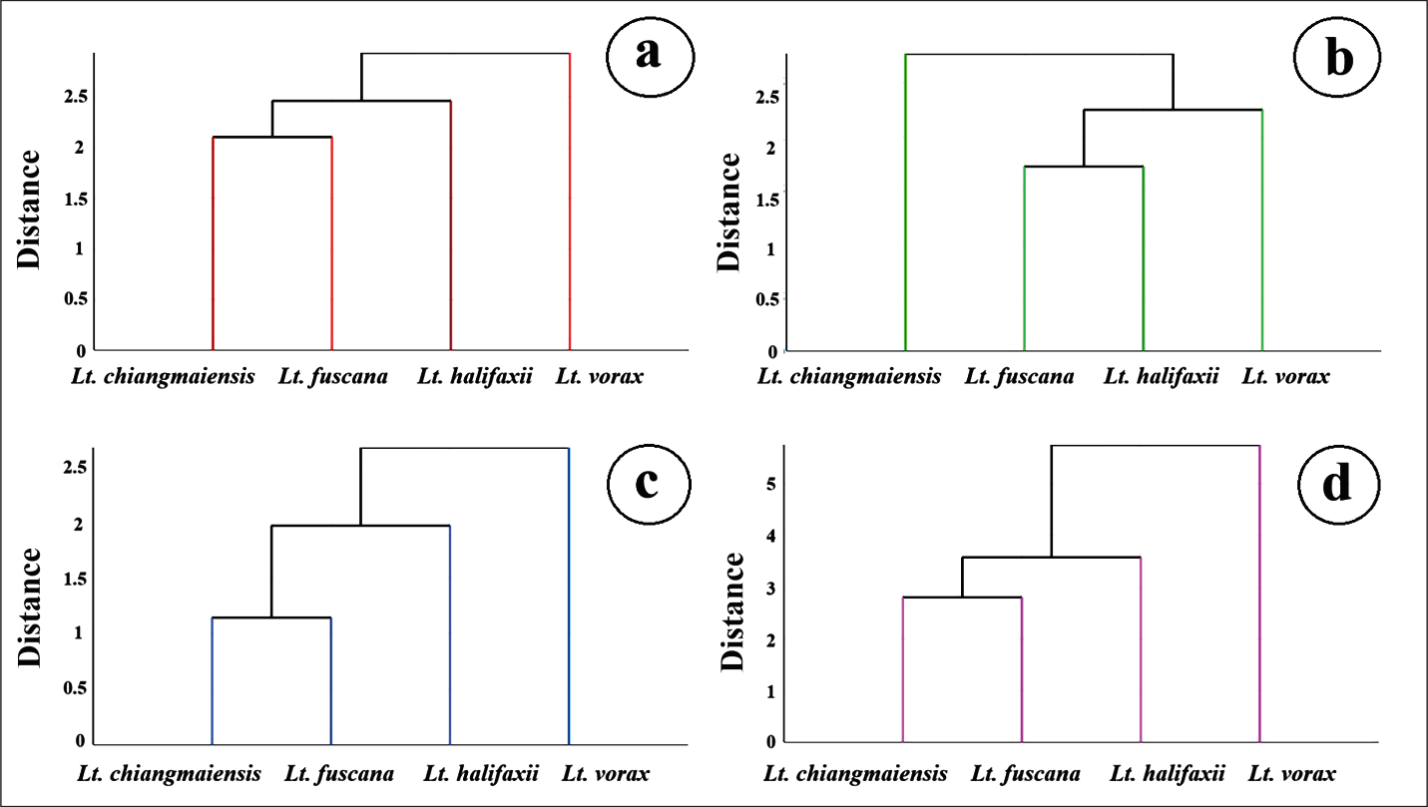
Unweighted Pair Group Method with Arithmetic Mean (UPGMA) trees derived from Mahalanobis distances illustrating the relationships among four *Lutzia* species based on four wing elements: (a) wing contour; (b) second submarginal cell; (c) first posterior cell; and (d) third posterior cell.

The cross-validated classification based on shape among the species revealed that the third posterior cell achieved the highest percentage of correct classifications, with an adjusted total assignment accuracy of 71%. This was followed by the wing contour at 49%, the second submarginal cell at 36%, and the first posterior cell at 29% (Table 5). The UPGMA trees (Fig. 6) illustrate the shape similarities among the species within each wing element.

**Table 5.** Percentage of correct identification based on the crossvalidated classification based on the Mahalanobis distance of four wing elements in four *Lutzia* species.

*Lutzia* species	No. of correctly assigned samples/ No. of total observed samples	Correct assignment scores (%)
Wing contour		
*Lt. chiangmaiensis*	15/30	50.00
*Lt. fuscana*	29/41	70.73
*Lt. halifaxii*	22/35	62.86
*Lt. vorax*	14/22	63.64
Adjusted total assignment accuracy		49
Second submarginal cell		
*Lt. chiangmaiensis*	20/30	66.67
*Lt. fuscana*	16/41	39.02
*Lt. halifaxii*	18/35	51.43
*Lt. vorax*	14/22	63.64
Total correct assignment score	68/128	53.13
Adjusted total assignment accuracy		36
First posterior cell		
*Lt. chiangmaiensis*	8/30	26.67
*Lt. fuscana*	26/41	63.41
*Lt. halifaxii*	11/35	31.43
*Lt. vorax*	16/22	72.73
Total correct assignment score	61/128	47.66
Adjusted total assignment accuracy		29
Third posterior cell		
*Lt. chiangmaiensis*	19/30	63.33
*Lt. fuscana*	36/41	87.80
*Lt. halifaxii*	26/35	74.29
*Lt. vorax*	20/22	90.91
Total correct assignment score	101/128	78.91
Adjusted total assignment accuracy		71

For the wing contour, the first posterior cell, and the third posterior cell, *Lt*. *chiangmaiensis* and *Lt*. *fuscana* showed more similarity in shape than with *Lt*. *halifaxii*, while *Lt*. *vorax* was the most distinct in shape from any other species. In contrast, within the second submarginal cell, *Lt*. *halifaxii* and *Lt*. *fuscana* are more similar to each other than to *Lt*. *vorax*, while *Lt*. *chiangmaiensis* appears most distinct from the other species.

## Discussion

Recent studies on mosquito classification emphasize the importance of integrating alternative techniques with standard morphological methods to enhance the accuracy of mosquito species identification [30,32,33]. GM, recognized for its low cost, has proven to be invaluable for field applications [34, 35-37], and mosquito wings have been validated as distinctive features suitable for this technique [20]. Additionally, recent research has identified wing cells, a substructure of the wing, as carrying taxonomic signals across various mosquito genera [23,25]. These findings are especially valuable for GM techniques when the entire wing of the specimen is incomplete, allowing the use of in-wing cell examination as a substitute. Our study is the first to identify a taxonomic signal in the wing cells of *Lutzia* species by using an outline-based GM approach.

Allometry describes the relationship between the size of an organism or its parts and the growth of its various parts or processes [38]. In this study, we investigated whether size influences the shape of four wing elements in *Lutzia* mosquitoes. Our results indicate that all four wing elements have a negative correlation between size and shape. However, the coefficients comparison indicated that the impact of size on shape in the three wing substructures ranged closely from 11.6% to 14.4%, while the wing contour exhibited a markedly lower effect at 4.9%. Given the significant implications of allometry in evolution, it is plausible that all wing elements may acquire species-specific identities but respond differently depending on the part of the wing structure. This aligns with findings from previous studies on several mosquito genera from Thailand, including *Aedes, Anopheles, Culex, Armigeres*, and *Mansonia*, which demonstrated that allometric relationships can vary among different wing elements [25,37]. Furthermore, the direction and magnitude of these allometric relationships may differ within the same mosquito species across different environmental conditions. This variability indicates that size, a trait highly susceptible to environmental influences, plays a critical role in shaping the morphological adaptations of mosquito populations [39,40].

The analysis of the size of the four wing elements studied herein indicated that *Lt*. *vorax* was significantly larger than the other species, while *Lt*. *chiangmaiensis*, *Lt*. *fuscana*, and *Lt*. *halifaxii* had very similar sizes, resulting in very low total correct assignment scores from the cross-validated classification based on size variables. Size is highly sensitive to environmental conditions and frequently overlaps between species [40]. Wing size was ineffective in distinguishing mosquito species within *Aedes*, *Anopheles*, *Culex*, and *Mansonia* [17,24,35,41]. Our findings confirm that the size of the wing cells is not a reliable factor for identifying *Lutzia* mosquitoes with the GM technique.

In mosquito species classification using modern techniques, size-based methods generally yield lower accuracy compared to shape-based approaches. For example, a previous study employing the scale-invariant feature transform algorithm—a traditional method that relies on handcrafted features—achieved a maximum classification accuracy of 82.4% [42]. In contrast, deep learning techniques, which analyze more complex morphological patterns beyond size, have shown significantly greater accuracy. One study employing a residual network with data augmentation reported 95.5% accuracy in mosquito species classification [42], and another using vision transformers achieved an accuracy of 99.6% [43]. These comparisons highlight the limitations of size-based methods and underscore the efficacy of advanced machine learning techniques for improving mosquito species classification.

Wing shape is under a stronger genetic signal than wing size, and our study results support this assertion [14]. The total correct assignment scores based on wing element shape analyses were significantly higher than those based on wing element size, with scores ranging from 29% to 71% for shape compared to 12%–19% for size. Among the four *Lutzia* species, the third posterior cell exhibited the highest classification accuracy. This observation was further supported by discriminant analyses, which demonstrated the least overlap between species groups, especially highlighting that *Lt*. *vorax* did not overlap with other groups within the third posterior cell. Similarly, a previous landmark-based GM analysis reported that *Lt*. *vorax* had the most distinctive overall wing shape compared to *Lt*. *chiangmaiensis, Lt*. *fuscana*, and *Lt*. *halifaxii* [7], aligning well with our findings for the third posterior cell. This consistency suggests that the outline-based GM approach focusing on the third posterior cell effectively supports classification of *Lutzia* species, particularly by distinguishing *Lt*. *vorax* from other species within the genus. Our results are also in agreement with Laojun et al. [25,37], who identified the third posterior cell as the most effective wing element for discriminating among species within the genera *Aedes, Anopheles*, and *Armigeres,* achieving classification efficiencies of 93.20%, 84.58%, and 82.61%, respectively.

Using UPGMA trees, the examination of wing element shape relationships among the four *Lutzia* species revealed similar patterns in the wing contour, the first posterior cell, and the third posterior cell, while a different pattern was observed in the second submarginal cell. The similar clustering patterns observed in the wing contour, first posterior cell, and third posterior cell suggest these wing structures may share comparable genetic or developmental influences, reflecting consistent evolutionary divergence among the species. In contrast, the unique clustering of the second submarginal cell indicates potential differences in genetic factors or developmental constraints, possibly due to functional adaptations specific to this wing region. Additionally, the patterns identified in our study differ from those found in a previous analysis that used the landmark-based GM approach in the same *Lutzia* species [7]. These pattern differences in wing cells and overall wing shape reflect the varying efficiency of species classification for each wing element by the GM technique.

A limitation of this study was the relatively small sample size, due to the low abundance of these mosquitoes in the field. Additionally, our analysis was limited to only the right wing. Future research should therefore include both wings to evaluate potential asymmetry, which could further enhance classification accuracy.

## Conclusion

In this study, we detected a taxonomic signal in the third posterior cell of the *Lutzia* mosquito species by using the outline-based GM approach. However, the effectiveness of this method for classifying *Lutzia* species is not yet considered excellent. Therefore, we recommend using the landmark-based GM approach as the primary method for classifying *Lutzia* mosquito species. In cases where specimen wings are damaged and unsuitable for landmark-based GM, the outline-based GM approach for analyzing wing-cell shape provides a practical alternative. This technique can be combined with standard morphological and molecular techniques to improve identification accuracy. Additionally, future research should explore advanced methods, such as deep learning approaches, to further enhance the accuracy of mosquito species classification.

## List of abbreviations

°C, degree Celsius

ANOVA, analysis of variance

cm, centimeter

DA, discriminant analysis

GM, geometric morphometrics

h, hour

mm, millimeter

NEF, normalized elliptic Fourier

PCA, principal component analysis

PCs, principal components

SIFT, the scale-invariant feature transform

UPGMA, Unweighted Pair Group Method with Arithmetic Mean

XYOM, XY Online Morphometrics.
